# Geographical and ethnic distribution of single nucleotide polymorphisms within genes of the folate/homocysteine pathway metabolism

**DOI:** 10.1007/s12263-014-0421-7

**Published:** 2014-08-09

**Authors:** Aristea Binia, Alejandra V. Contreras, Samuel Canizales-Quinteros, Victor Acuña Alonzo, M. Elizabeth Tejero, Irma Silva-Zolezzi

**Affiliations:** 1 Nutrition and Health Research, Nestlé Research Center, Lausanne, Switzerland; 2 Laboratorio de Nutrigenética y Nutrigenómica, Instituto Nacional de Medicina Genómica (INMEGEN), Mexico City, Mexico; 3 Unidad de Genómica de Poblaciones Aplicada a la Salud, Facultad de Química, Universidad Nacional Autónoma de México (UNAM)-INMEGEN, Unidad de Biología Molecular y Medicina Genómica, Instituto Nacional de Ciencias Médicas y Nutrición “Salvador Zubirán” (INCMNSZ), Mexico City, Mexico; 4 Escuela Nacional de Antropología e Historia (ENAH), Molecular Genetics Laboratory, Mexico City, 14030 Mexico

**Keywords:** Folate, Homocysteine, *MTHFD1* G1958A, *MTRR *A66G, *MTHFR* A1298C, *MTHFR* C677T

## Abstract

High levels of plasma homocysteine are associated with an increased risk of many health conditions influenced by both environmental and genetic factors. The objective of this study was to provide the geographical distribution of folate pathway genetic polymorphisms in Mexico and the comparison with the reported frequencies in different continental populations. This study included the analysis of the genotypic frequencies of eight polymorphisms in genes of the folate/homocysteine metabolic pathway in 1,350 Mestizo and Amerindian subjects from different regions in Mexico and 836 individuals from European, African and Asian populations of the 1,000 Genomes Project. In Mexican Mestizo and Amerindian populations, the *MTHFR* C677T risk genotype (TT) was highly prevalent (frequency: 25 and 57 %, respectively). In Mestizos, the frequency showed clear regional variation related to ancestry; the Guerrero subpopulation with the highest Amerindian contribution had the highest TT frequency (33 %). The *MTHFD1* G1958A AA risk genotype was also enriched in Mexican Mestizos and Amerindians (frequency: 34 and 58 %, respectively), whereas in African and Asian ancestry populations the frequency for AA was low (~4 %). All together risk genotypes showed regional differences, and Sonora had significantly different genetic frequencies compared with the other regions (*P* value <0.05). Our study illustrates differential geographical distribution of the risk variants in the folate/homocysteine metabolic pathway relative to ethnic background. This work supports that certain areas of the world have increased needs for folic acid and vitamin B supplementation, and this information needs to be considered in public health guidelines and eventually policies.

**Electronic supplementary material:**

The online version of this article (doi:10.1007/s12263-014-0421-7) contains supplementary material, which is available to authorized users.

## Introduction

Folates are a group of molecules that belong to the vitamin B family, differing by their oxidation state, number of glutamic acid moieties and one-carbon substitutions (Forges et al. 2007). They are part of the commonly known folate-mediated one-carbon metabolism pathway, a system consisting of a number of interdependent metabolic cycles, that are necessary in a large number of biochemical processes, such as amino acid metabolism, purine and pyrimidine synthesis and methylation of nucleic acids, proteins and lipids (Forges et al. 2007; Carr et al. 2009). The folate-mediated one-carbon metabolism pathway is essential for human health and development, which is made evident by the fact that folate deficiency has been linked to a variety of conditions along the life span including adverse pregnancy outcomes such as neural tubes defects (NTDs). Hyperhomocysteinemia a known risk factor to a range of conditions, such as cardiovascular and neurodegenerative disorders, has also been associated with this metabolic pathway (Forges et al. 2007). Folic acid supplementation during the periconceptional period reduces greatly the risk of NTDs (Prevention of neural tube defects: results of the Medical Research Council Vitamin Study. MRC Vitamin Study Research Group 1991), and many countries have now mandatory fortification legislation and programs in place to avert the disease risks linked to folate deficiencies (Wolff et al. 2009; Berry et al. 1999). In Latin America, following the implementation of folic acid fortification, NTDs prevalence was decreased from 33 to 59 % (Rosenthal et al. 2013). However, the benefit of folic acid supplementation in reducing the risk for cardiovascular diseases (CVD) by decreasing the homocysteine levels is not clear, revealing a more complex relationship between vitamin intake levels, homocysteine and CVD (Bonaa et al. 2006; Homocysteine Studies 2002; Toole et al. 2004; Lonn et al. 2006).

**Fig. 1:**
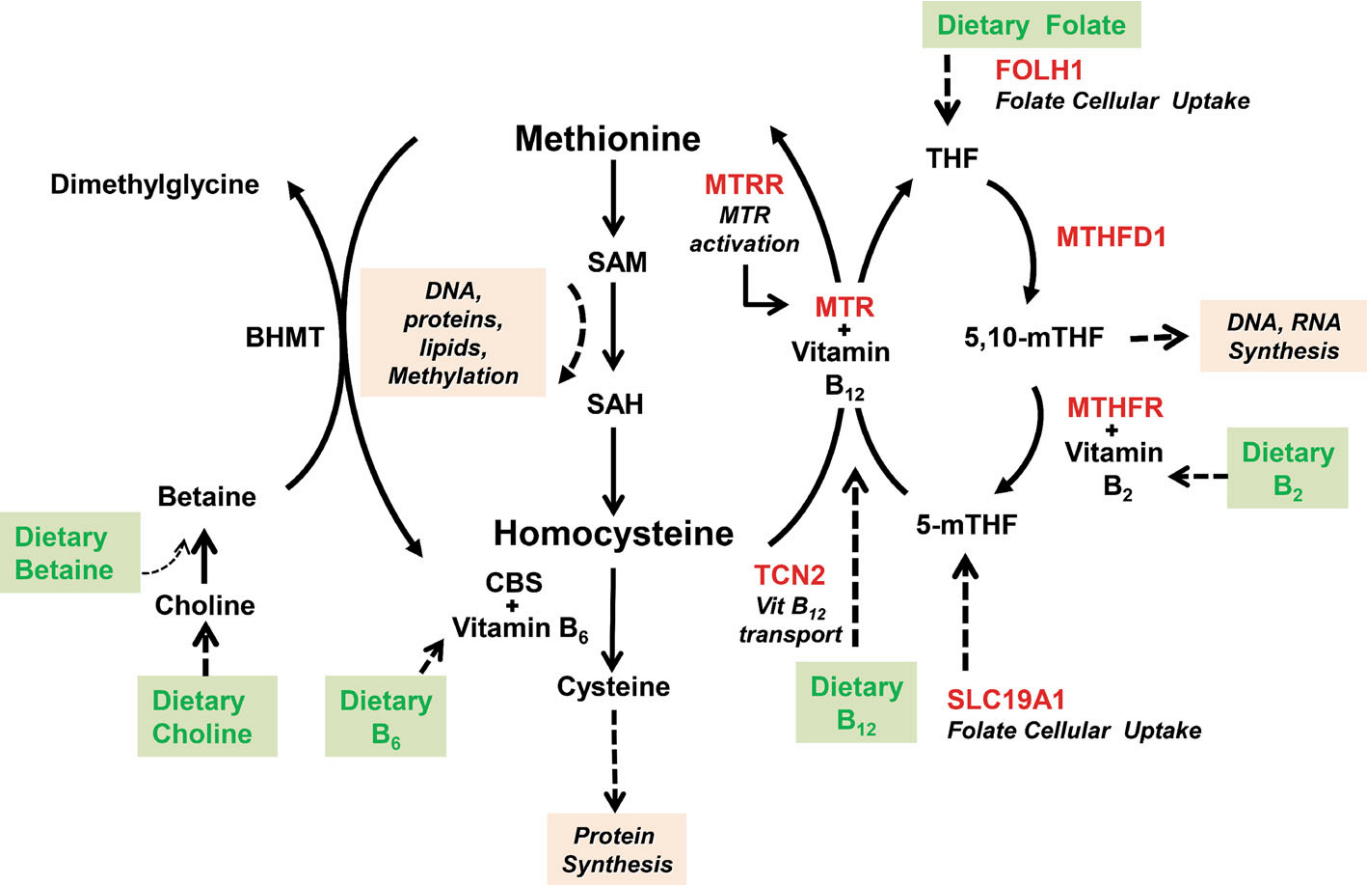
Metabolic cycle of homocysteine, the seven enzymes carrying the functional SNPs analyzed in the study are shown in red (*SAM* *S*-Adenosyl methionine, *SAH* *S*-Adenosyl-l-homocysteine, *BHMT* Betaine-homocysteine S-methyltransferase, *CBS* Cystathionine beta synthase, *FOLH1* Folate hydrolase, *MTHFD1* Methylenetetrahydrofolate dehydrogenase 1, *MTHFR* Methylenetetrahydrofolate reductase, *MTRR* Methionine synthase reductase, *MTR* Methionine synthase, *SLC19A1* Solute carrier family 19, *TCN2* Transcobalamin II)

Several studies have identified associations between polymorphisms in genes related to the folate pathway (Fig. 1), some of them with known functional implications, and specific conditions, including NTDs, CVD, psychiatric disorders and some types of cancers, such as colorectal, pancreatic, gastric and ovarian (Berry et al. 1999; Greene et al. 2009; Gueant-Rodriguez et al. 2006; Fredriksen et al. 2007; Koushik et al. 2006; Lissowska et al. 2007). However, there is evidence that shows no effects or conflicting findings about these associations of genetic variants of folate metabolism (Ding et al. 2013; Stevens et al. 2008; Sharp and Little 2004; Zhou et al. 2012; Yu et al. 2010). One problem in the meta-analyses of genetic association studies is the scarcity of data sets that would allow for the analysis of covariates (Jennings and Willis 2014); consequently, the metabolic effects associated with altered one-carbon metabolism of these diseases require extensive investigations.

In relation to polymorphisms in genes functionally related to this pathway, as of today, only some have been analyzed in case–control and/or in epidemiological studies in different populations. Some of these extensively studied are within the methylenetetrahydrofolate reductase gene (*MTHFR*), which encodes for an enzyme catalyzing the irreversible reduction of 5, 10-methylenetetrahydrofolate to 5-methyltetrahydrofolate serving as the methyl donor for the vitamin-B_12_-dependent remethylation of homocysteine (Hcy) to methionine (Fig. 1) (Frosst et al. 1995). These polymorphisms are the *MTHFR* C677T (A222V, rs1801133) and A1298C (E429A, rs1801131), both known to result in a decreased activity of the enzyme (Frosst et al. 1995; Weisberg et al. 1998). In addition to these functional polymorphisms, other genetic variants within coding regions of related genes in this pathway have been found to be associated with a variety of complex traits and disorders, including the methionine synthase reductase *MTRR* A66G, the methionine synthase or 5-methyltetrahydrofolate-homocysteine methyltransferase *MTR* A2756G, the methylenetetrahydrofolate dehydrogenase 1 *MTHFD1* G1958A, the folate hydrolase *FOLH1* T484C, the solute carrier family 19 (folate influx transporter) member 1 *SLC19A1* A80G and the transport protein of vitamin B_12_, transcobalamin II *TCN2* C776G (Gaughan et al. 2001; Lissowska et al. 2007; Silva et al. 2011; Guo et al. 2013; Xie et al. 2012; Dervieux et al. 2004b; Laverdiere et al. 2002; Martinelli et al. 2006; Moskau et al. 2007; Parveen et al. 2013). The frequencies of some of these polymorphisms are known to vary across different geographical regions and ethnic groups. For example, the *MTHFR* 677TT genotype frequency is often reported to be high in European, Asian, Central and South American (10–32 %), low in different African populations (0–3 %) (Wilcken et al. 2003) and also showing geographical gradients among Chinese Han populations (Yang et al. 2013). The possibility of having accurate information about the frequency of these polymorphisms and better understanding of their variation between and within populations can help experts in this field improve the design of association studies, aid in the evaluation of regional dietary requirements and inform public health policy makers.

The Mexican population is mainly composed by Mestizos, who are individuals with a genetic background consisting of Amerindian, European and, to a lesser extent, African ancestries, and as a result of admixture, the resulting linkage disequilibrium patterns in all these genetic loci may affect the allele frequencies (Silva-Zolezzi et al. 2009). This population has been reported to have one of the highest frequencies of the *MTHFR* 677T allele worldwide, above to that reported in some European and African populations (Wilcken et al. 2003), suggesting that this results due to an enrichment of this variant in Mexicans through the admixture process and the Amerindian ancestral contribution. To better understand the distribution of this and other variants in the folate-mediated one-carbon metabolism pathway in a highly admixed population such as Mexicans, we describe and compare the allele and genotype frequencies of *FOLH1* T484C, *MTR* A2756G, *MTHFD1* G1958A, *MTRR* A66G, *MTHFR* A1298C, *MTHFR* C677T, *SLC19A1* A80G and *TCN2* C77G (Fig. 1; Suppl. Table 1) in 1,104 Mexican Mestizos from the Mexican Genome Diversity Project (MGDP), 246 subjects with Amerindian ancestry and 836 samples from Phase I, 1,000 Genomes Project, from distinct continental locations, and/or ancestral contributions, including African, Asian and European.

## Methods

### Samples

**Fig. 2:**
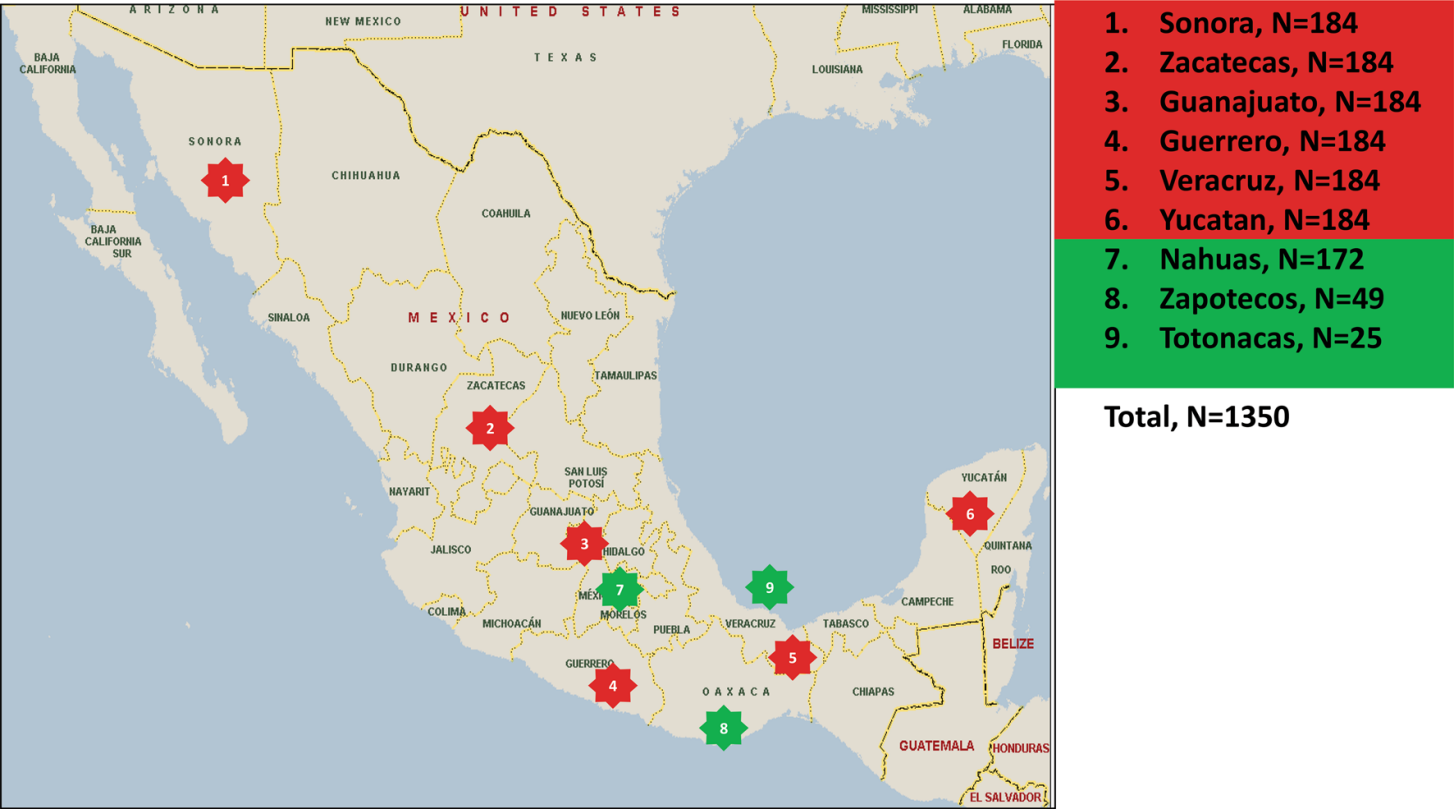
Geographical distribution of the Mestizos (MEX) and the three Amerindian (AMI) Mexican populations included in the study

DNA samples included in this study are from 1,104 adult Mexican Mestizos (MEX) from the Mexican Genome Diversity Project (MGDP) (Silva-Zolezzi et al. 2009), corresponding to 184 unrelated individuals (92 males and 92 females) from each of six different states of Mexico: Sonora (SON), Guanajuato (GUA), Zacatecas (ZAC), Guerrero (GUE), Veracruz (VER) and Yucatan (YUC). In addition, we included samples from three Amerindian groups (AMI), 172 Nahuas (NAH), 49 Zapotecos (ZAP) and 25 Totonacas (TOT) (Fig. 2). All individuals in the Amerindian groups, their parents and grandparents recognized themselves as indigenous, had been born and lived in their home communities and spoke their native language. This study was conducted according to the principles expressed in the Declaration of Helsinki. All the participants of the MGDP and the Amerindian groups signed a consent approved by the Scientific, Ethics and Biosafety Review Boards from the National Institute of Genomic Medicine (INMEGEN) and the National Institute of Medical Sciences and Nutrition Salvador Zubirán (INCMNSZ), respectively. The genotypic and allelic frequencies analysis of this study was approved by the Ethics committee as previously reported (Contreras et al. 2014; Silva-Zolezzi et al. 2009; Leon-Mimila et al. 2013). No phenotypic data of the subjects were available or analyzed. DNA was isolated from buffy coats obtained from blood using the QIAamp DNA Blood Maxi Kit (Qiagen GmbH, Hilden, Germany).

### Genotyping

We analyzed eight SNPs, *MTHFR* C677T and A1298C, *MTR* A2756G, *MTRR* A66G, *MTHFD1* G1958A, *TCN2* C776G, *FOLH1* T484C and *SLC19A1* A80G, by allelic discrimination with TaqMan^®^ SNP genotyping assays (Applied Biosystems, Life Technologies CA, USA), according to the manufacturer’s protocol. We used 15 ng of genomic DNA as template, the assays run on 7900HT PCR Real Time System (Applied Biosystems, Life Technologies CA, USA) and then analyzed using the Sequence Detection System v2.2.2. To verify the accuracy of the genotyping, we compared our data with the genotypes for all SNPs, except *MTRR* A66G, obtained for a subset of 296 individuals from the Mexican Mestizos group by using Illumina 550 K (Moreno-Estrada et al. 2014) and 510 K arrays (Illumina Inc., CA, USA).

### Genotype data from public databases

Individual genotype data, publicly available, from East Asian (ASIA), African (AFR) and European (EUR) participants in the 1000 Genomes Project, Phase I were retrieved from www.1000genomes.org. From East Asia, 97 Han Chinese from Beijing, 89 Japanese from Tokyo and 100 Southern Han Chinese were included. The African population consisted of 97 Luhya from Webuye, Kenya and 88 Yoruba from Ibadan, Nigeria. Finally, the European population included 89 British from England and Scotland, 93 Finnish, 98 Toscani from Italy and 85 Utah residents with Northern and Western European ancestry. Detailed information on the projects and the recruited subjects can be found in www.1000genomes.org (Genomes Project et al. 2010). For *MTHFR* C677T, genotyping data were retrieved in August 2013 from the ALlele FREquency Database http://alfred.med.yale.edu/alfred/index.asp and were used to visualize the world distribution of the risk genotype (Rajeevan et al. 2012).

### Statistical analysis

Allele and genotype frequencies were calculated by direct counting (Griffiths et al. 2000). Deviations from Hardy–Weinberg equilibrium (*P* < 0.005) and comparison of allele or genotype frequencies between different populations were tested with Fisher’s exact test. Calculated confidence intervals (95 %) around a completion rate were constructed in SPSS. Genotype distributions in different populations were compared using Kruskal–Wallis test. All maps were constructed using the MapPoint software (Microsoft MapPoint, version 2010).

## Results

All SNPs in the Mexican Mestizos (MEX) had call rates >95 %, and in the Amerindians (AMI) samples, three genotyping assays (*MTHFD1* G1958A, *MTHFR* A1298C, *MTR* A2756G) had a call rate of >97 % and five assays (*MTHFR* C677T, *MTRR* A66G, *SLC19A1* A80G, *TCN2* C77G) had a call rate of 89 % (Supp. Table 1). For all SNPs, genotypic frequencies were in accordance with Hardy–Weinberg Equilibrium (*P* > 0.005). The concordance between our frequency genotyping data obtained by allelic discrimination with TaqMan^®^, and those acquired from the Mexican Genome Diversity Project using Illumina 510 and 550 K arrays was high (=99.43 %).

### Geographical distribution of folate-mediated one-carbon metabolism pathway alleles and genotypes showing continental differences but not major intra-population differences in Mexican Mestizos

**Fig. 3:**
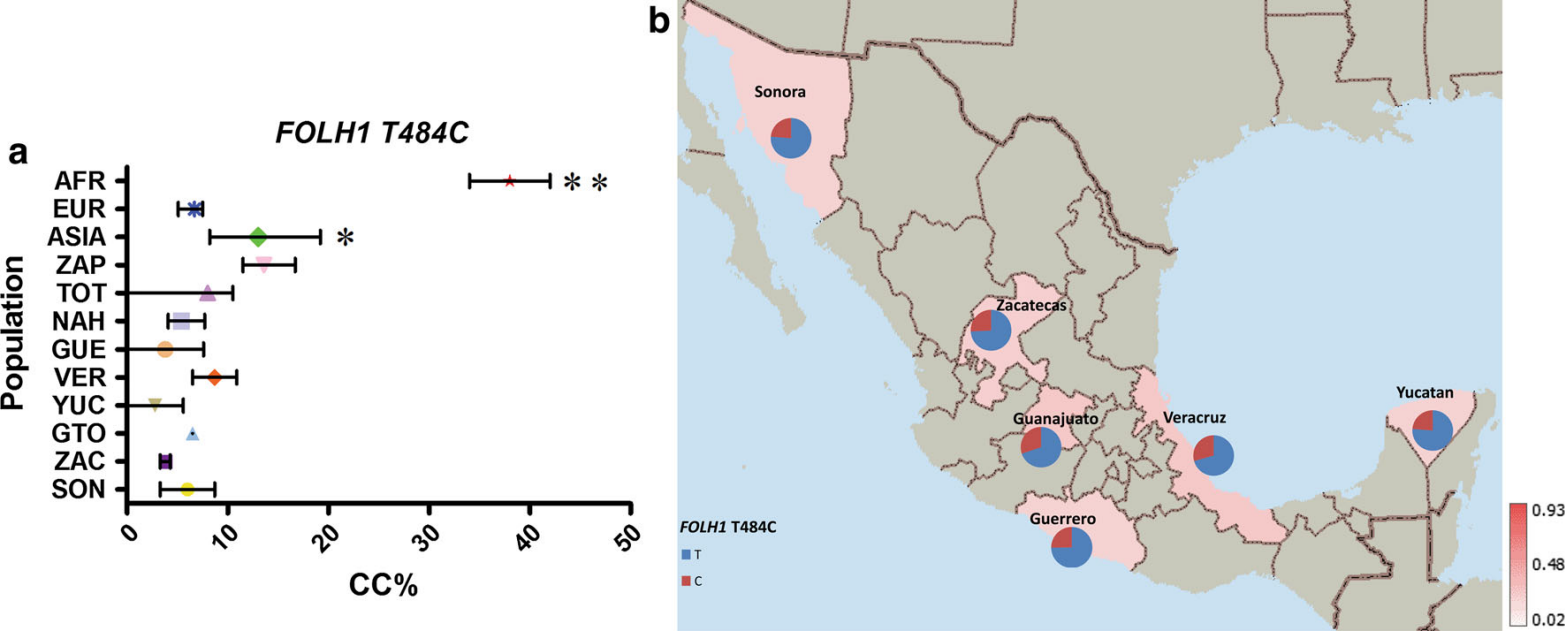
**a** Comparative frequency of the homozygotes for the risk allele of *FOLH* T484C in the studied populations (*P value >0.0001 and <0.05, **P value <0.0001 for significant differences in genotype frequencies), **b** minor allele frequency of *FOLH* T484C in six Mexican Mestizo groups

**Fig. 4:**
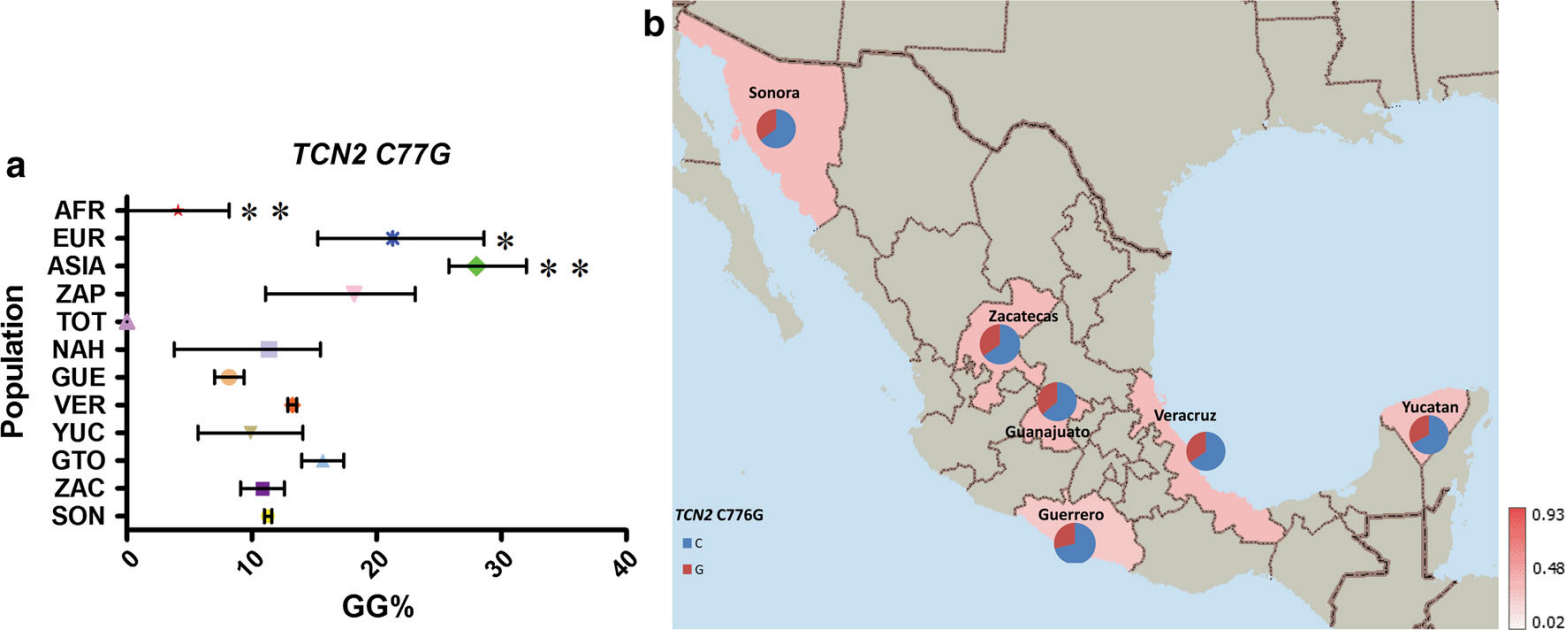
**a** Comparative frequency of the risk allele homozygotes for *TCN2* C77G in the studied populations (*P value >0.0001 and <0.05, **P value <0.0001 for significant differences in genotype frequencies), **b** minor allele frequency of *TCN2* C77G in six Mexican Mestizo groups

**Fig. 5:**
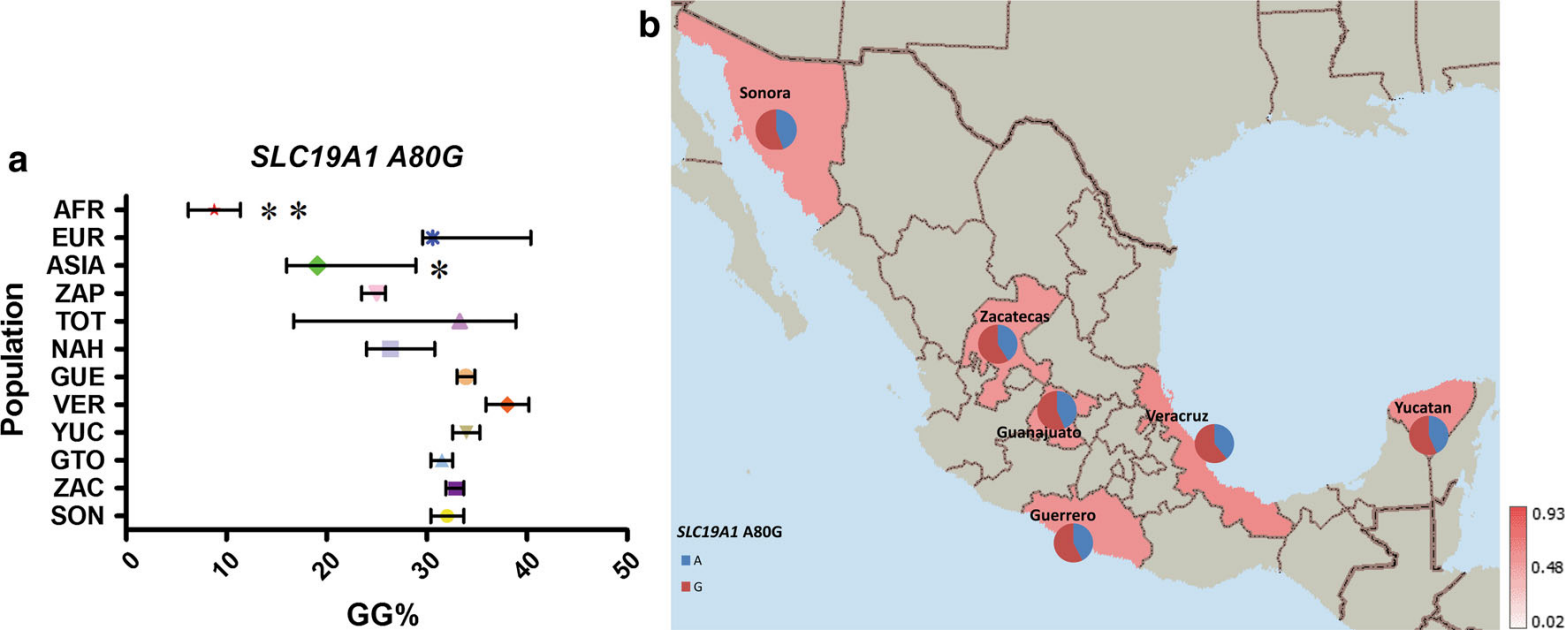
**a** Comparative frequency of the risk allele homozygotes for *SLC19A1* A80G in the studied populations (*P value >0.0001 and <0.05, **P value <0.0001 for significant differences in genotype frequencies), **b** minor allele frequency of *SLC19A1* A80G in the six Mexican Mestizo Groups

**Fig. 6:**
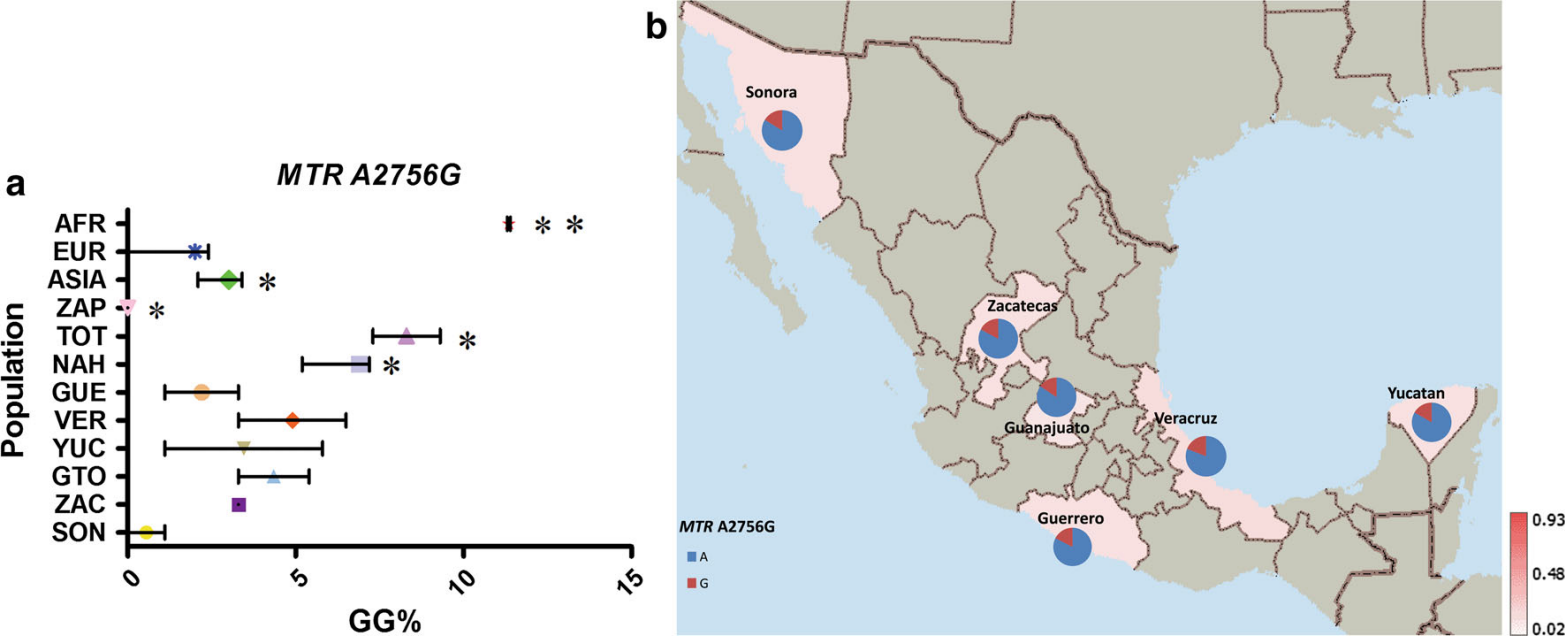
**a** Comparative frequency of the risk allele homozygotes for *MTR* A2756G in the studied populations (*P value >0.0001 and <0.05, **P value <0.0001 for significant differences in genotype frequencies), **b** minor allele frequency of *MTR* A2756G in six Mexican Mestizo groups

Four of the eight variants analyzed, *FOLH1* T484C, *TCN2* C77G, *SLC19A1* A80G and *MTR* A2756G, showed a similar average frequency distribution between MEX, AMI and EUR groups (MAF frequencies: 23–26, 31–43, 54–58 and 17–25 %, respectively), which correlates with either no significant differences or very low variation in their frequency within MEX groups (Figs. 3, 4, 5, 6).

The risk genotype (CC) for *FOLH1* T484C had a frequency ≤14 % in all Mexican groups (MEX, NAH, ZAP and TOT). EUR and ASIA had a CC frequency of 6 % and of 13 %, respectively. The highest CC frequency was observed in the AFR group (34 %) (Supp. Table 2; Fig. 3).

The risk genotype (GG) for *TCN2* C77G ranged from 8 to 18 % in all Mexican populations (MEX and AMI), excluding TOT in which the GG genotype was absent. The frequency of the GG genotype was higher in EUR (20 %) and ASIA (29 %) than in AFR (6 %), where it had the lowest frequency of all continental groups (Supp. Table 3; Fig. 4).

The risk genotype (GG) for *SLC19A1* A80G had a high frequency in MEX, AMI and EUR populations, ranging from 25 % in ZAP to 38 % in VER (Supp. Table 4; Fig. 5). ASIA had 21 % of the GG genotype and AFR showed the lowest frequency (9 %) (Supp. Table 4; Fig. 5).

Finally, the risk genotype (GG) for *MTR* A2756G was ≤11 % in most populations included (Supp. Table 5; Fig. 6). EUR populations and MEX populations with high European ancestry, SON and GUE had the lowest frequencies for the *MTR* A2756G (≤2 %). In ZAP, there was no carrier of the *MTR* A2756G GG genotype. AFR had the highest frequency of the GG genotype for this SNP (11 %).

### Geographical distribution of folate-mediated one-carbon metabolism pathway alleles and genotypes showing continental variation and intra-population differences in Mexican Mestizos

In contrast to the additional four out of the eight variants analyzed, *MTRR* A66G, *MTHFR* C677T, A1298AC and *MTHFD1* G1958A showed a gradient in the frequency between MEX groups and highly significant differences between AMI and EUR groups (*P* values <10^−4^).

**Fig. 7:**
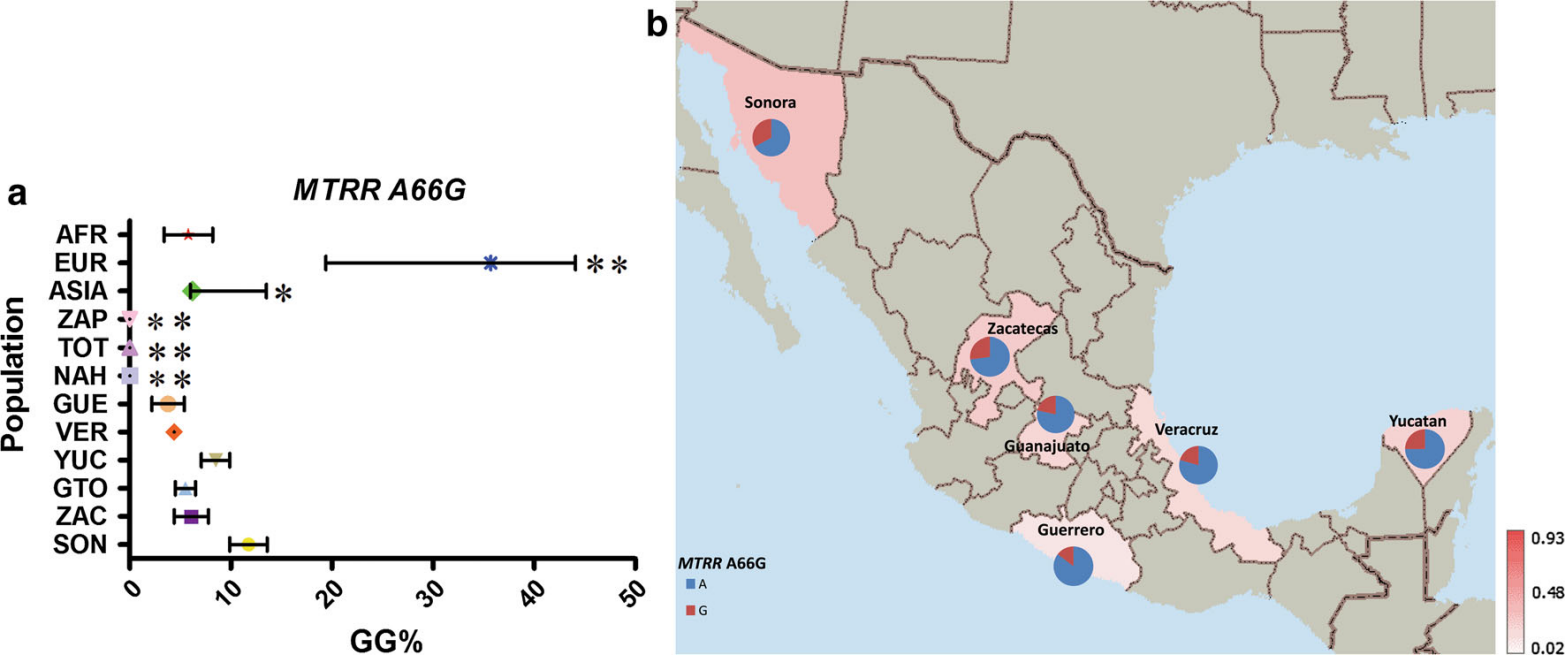
**a** Comparative frequency of the risk allele homozygotes for *MTRR* A66C in the studied populations (*P value >0.0001 and <0.05, **P value <0.0001 for significant differences in genotype frequencies), **b** minor allele frequency of *MTRR* A66C in six Mexican Mestizo groups

The risk genotype (GG) for *MTRR* A66G presented the highest frequency in EUR (33 %), deviating from ASIA, AFR, AMI and MEX (Supp. Table 6; Fig. 7). Among MEX, the group with the highest European ancestry (SON) had the highest frequency of GG genotype carriers (Fig. 7). In EUR, we observed a North-to-South gradient in the GG genotype frequencies, with Finnish having the highest frequency (44 %), followed by British (32 %) and Italians having the lowest of the three (19 %) (data not shown).

**Fig. 8:**
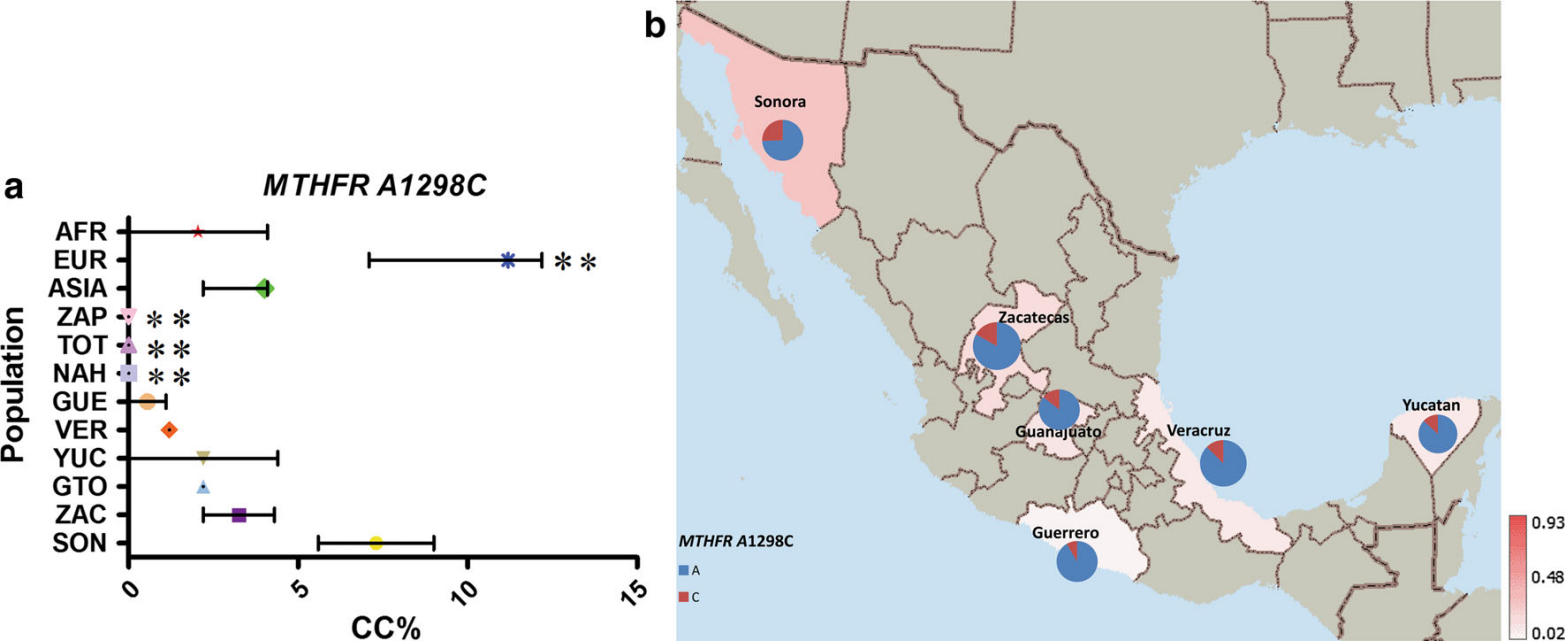
**a** Comparative frequency of the risk allele homozygotes for *MTHFR* A1298C in the studied populations (*P value >0.0001 and <0.05, **P value <0.0001 for significant differences in genotype frequencies), **b** minor allele frequency of *MTHFR* A1298C in six Mexican Mestizo groups

The risk genotype (CC) for the *MTHFR* A1298C presented a similar distribution as for *MTRR* A66G. Overall, the frequency of CC genotype was ≤11 %, and EUR and SON within the MEX groups had the highest frequencies, 11 and 7 %, respectively (Supp. Table 7; Fig. 8). In contrast to *MTRR* A66G, European populations from the 1,000 GP had similar risk genotypes frequencies for *MTHFR* A1298C (data not shown).

**Fig. 9:**
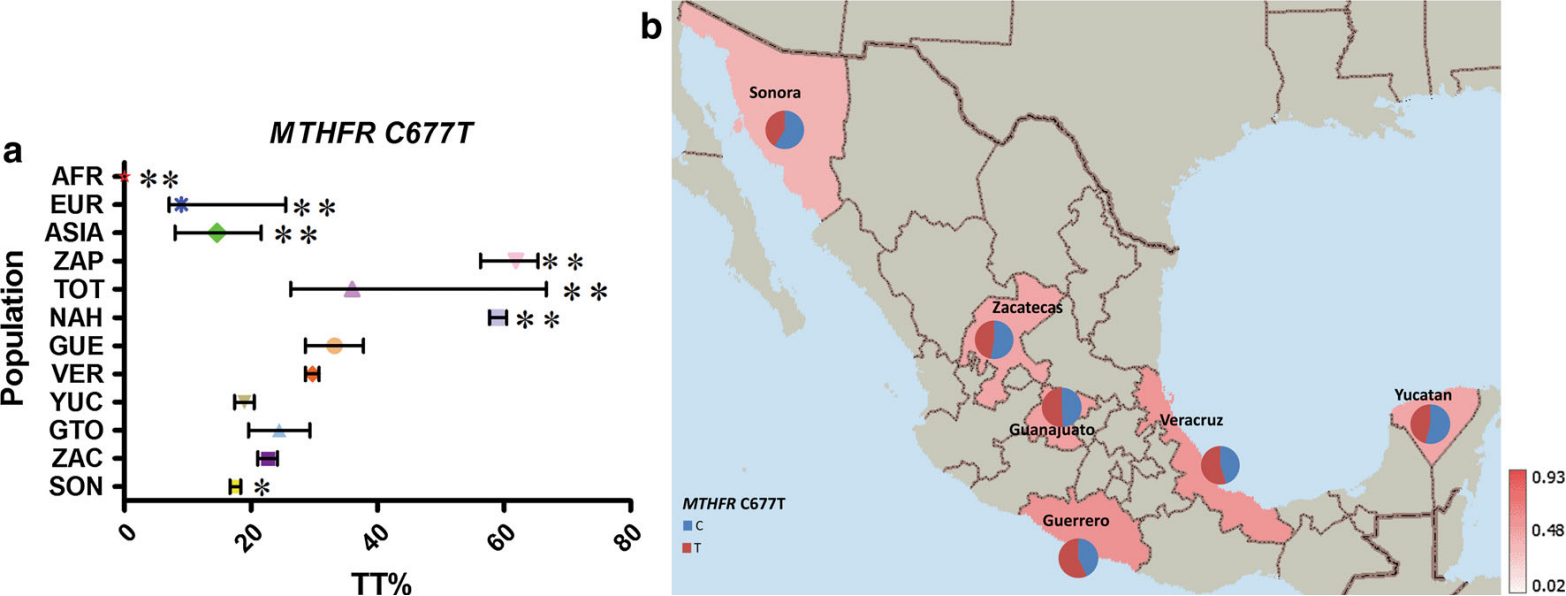
**a** Comparative frequency of the risk allele homozygotes for *MTHFR* C677T in the studied populations (*P value >0.0001 and <0.05, **P value <0.0001 for significant differences in genotype frequencies), **b** minor allele frequency of *MTHFR* C677T in six Mexican Mestizo groups

**Fig. 10:**
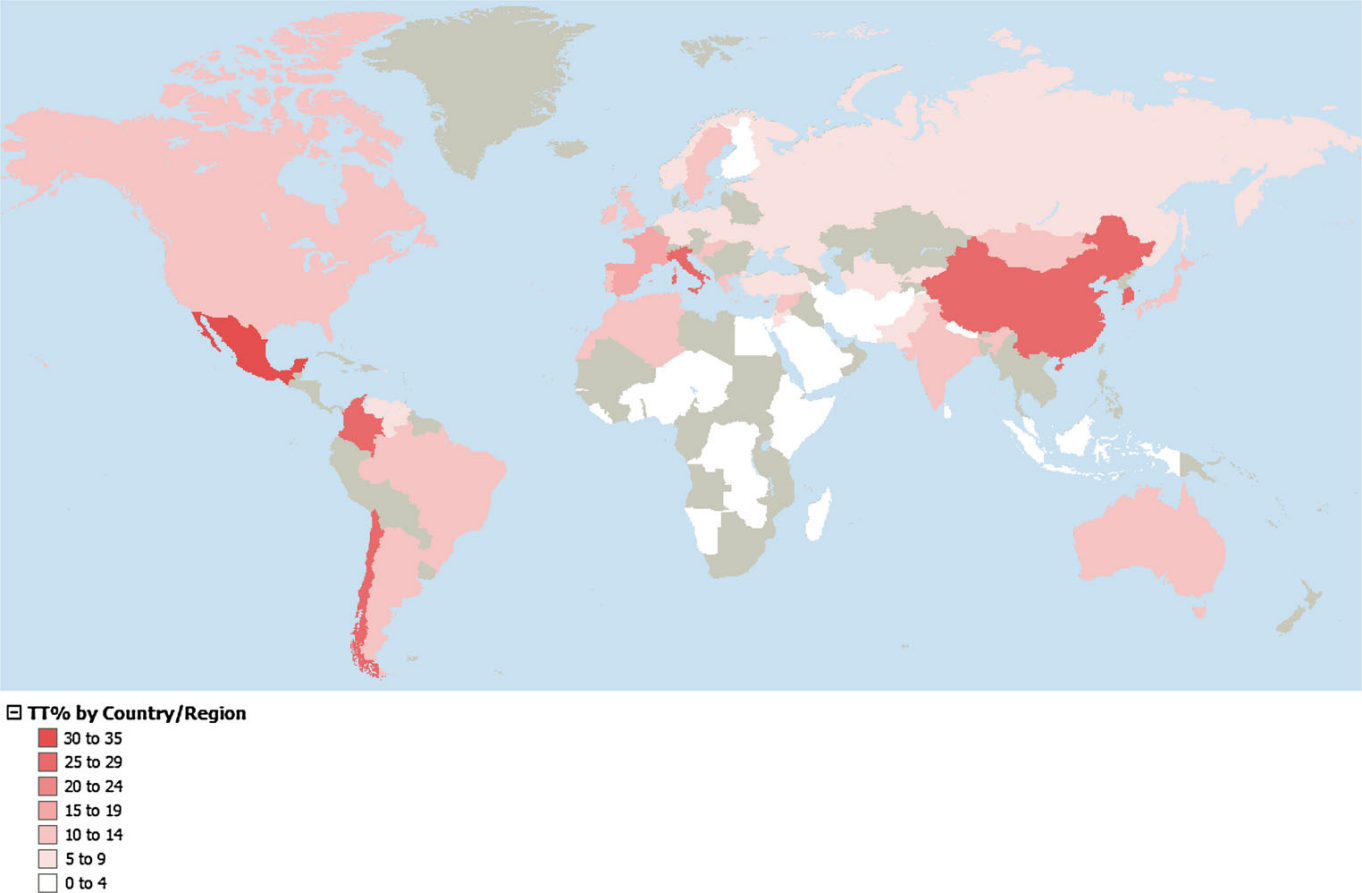
Global distribution of TT % for *MTHFR* C677T (Source: ALFRED database)

In *MTHFR* C677T, homozygotes for the risk T allele were found in very high frequency in all three AMI groups included in the study. NAH and ZAP had a frequency of subjects with a TT genotype of 59 and 62 %, whereas in TOT 36 % had a TT genotype (Supp. Table 8; Fig. 9). In AFR populations, there was no TT genotype, whereas ASIA and EUR had a 15 and 13 %, respectively. In the MEX groups, the TT frequency ranged from 17 % in SON to 33 % in GUE. The risk allele had an average frequency of 50 % frequency in MEX (Supp. Table 8; Fig. 9). Genotyping data for *MTHFR* C677T retrieved from ALFRED database (including studies by Botto et al., Wilcken et al. and Yang et al.) revealed a similar world distribution to that of the 1,000 GP populations we included in our study (Botto and Yang 2000; Wilcken et al. 2003; Yang et al. 2013), excepting China which presented a lower frequency in 1000GP population that the one of previous publications (Fig. 10) (Yang et al. 2013).

**Fig. 11:**
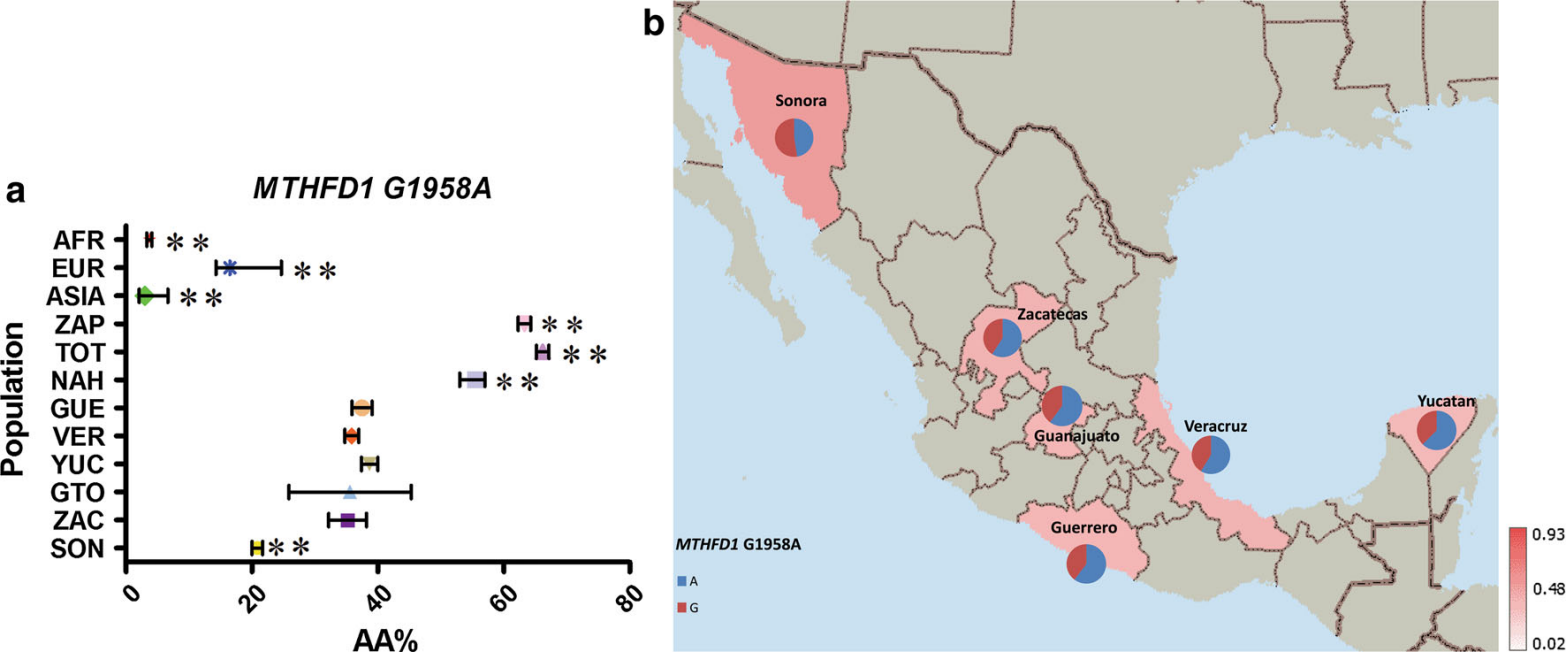
**a** Comparative frequency of the risk allele homozygotes for *MTHFD1* G1958A in the studied populations (*P value >0.0001 and <0.05, **P value <0.0001 for significant differences in genotype frequencies), **b** minor allele frequency of *MTHFD1* G1958A in six Mexican Mestizo groups

The risk genotype (AA) for *MTHFD1* G1958A was found more frequent in all AMI (55–65 %), whereas in ASIA and AFR the frequency was low (4 %). EUR and SON had similar frequency, 19 and 21 %, respectively (Supp. Table 9). The rest of the MEX and AMI have an intermediate distribution of the homozygous carriers for the risk allele ranging from 35 to 39 %, with SON differing significantly (*P* value <0.0001) from the rest (Fig. 11).

### Gender differences in the genotypes distribution in Mexican Mestizos

We examined whether the genotypes distribution for the eight SNPs were significantly different between males and females in the MEX populations. We only found a significant difference in the genotype distribution between genders for *FOLH1* T484C (*P* = 0.0006). In women, MAF of *FOLH1* T484C was higher than in men (Suppl. Table 10; Suppl. Fig. 1). For *SLC19A1* A80G, *MTHFD1* G1958A, *MTHFR* A1298C and *MTR* A2756G, borderline significant differences were found (Suppl. Table 1).

## Discussion

Our study investigated the distribution of eight disease risk-associated alleles and genotypes of functional SNPs within seven genes encoding for enzymes from the folate pathway in both, admixed (MEX) and indigenous (AMI) Mexican populations, and a comparative analysis was done against their frequencies in samples from major continental population groups: European (EUR), East Asian (ASIA) and Africans (AFR).

Some risk genotypes frequencies were very similar among and between MEX and AMI groups, including *FOLH1* T484C, *TCN2* C77G and *MTR* A2756G. Of these, *TCN2* C77G showed continental differences, and *FOLH1* T484C, *MTR* A2756G and *SLC19A1* A80G had similar frequencies in most populations analyzed, only showing a significantly different frequency in AFR groups. In contrast, the risk genotypes for *MTRR* A66C, *MTHFR* A1298C, *MTHFR* C677T and *MTHFD1* G1958C variants showed both continental and intra-population differences in MEX and AMI groups (*P* < 0.0001). In the three AMI groups included in our study, no *MTRR* 66CC or *MTHFR* 1298CC genotypes were found, and a low frequency of the risk allele for both was observed (MAF ≤ 7 %). These risk genotypes were more frequent in EUR (33 and 11 %, respectively), and among the MEX groups had the highest frequency in SON, in relation to the high European ancestral contribution to this population group. The risk-associated genotypes *MTHFD1* 1958AA and *MTHFR* 677TT had a particularly high frequency in the MEX population, 34 and 25 %, respectively. The frequencies of both SNPs in the AMI groups included in our study were >55 %, supporting the hypothesis that this phenomenon is related to the Amerindian ancestral contribution to the admixture process (Wilcken et al. 2003; Davalos et al. 2000; Juarez-Velazquez et al. 2010). This is further supported by the observed gradient in the frequency of these variants in the MEX populations analyzed, within which, the highest and the lowest frequencies were observed in GUE and SON, the groups with, respectively, the highest and the lowest Amerindian ancestry contribution in this set of samples (Silva-Zolezzi et al. 2009). Interestingly, for these SNPs, the frequencies of the risk-associated genotype were lower in ASIA and EUR populations (<20 %) and almost absent in the AFR populations (≤4 %), which underlines the more specific relevance of these genetic variants for populations with Amerindian ancestry, such as most populations of central and south America, Native American and most Hispanic groups living in the USA.

Our observed *MTHFR* C677T frequencies were similar to those of populations with the same ancestry of the 1000GP or of previous reports (Davalos et al. 2000; Juarez-Velazquez et al. 2010; Wilcken et al. 2003). Nevertheless, we have provided a more detailed description of within population differences that was not captured in any of the previous studies. Our results are in agreement with a previous study investigating the prevalence of *MTHFR* 677TT genotype in different regions in Mexico in which the lowest frequency was observed in the north of the country (in our study SON) and the highest frequency in the center of the country (in our study GUE) (Mutchinick et al. 1999). In the same study, Mutchinick et al. reported highly significant differences of *MTHFR* 677TT genotype frequency in Mexico compared with other countries, supporting that the high prevalence of NTDs in Mexico may be related to the particularly frequent presence of the non-functional T allele in the population. Indeed, in Mexico, the highest prevalence of NTDs in the world has been documented. According to the data from the program of Registry and Epidemiological Surveillance of External Congenital Malformations (RYVEMCE), approximately one in 250 conceptions that reach 20 weeks of gestation or more present a NTD. This number is three or four times higher than that observed in populations from South America and Spain (Mutchinick et al. 1988, 1999; Conjoined twins–an epidemiological study based on 312 cases. The International Clearinghouse for Birth Defects Monitoring Systems 1991). The prevalence at birth for anencephaly and spina bifida, the NTDs most frequently observed, was 18.4 and 15.6 per 10,000 births, respectively (Mutchinick et al. 1988, 1999). Interestingly, the state of Guerrero (GUE) has shown a trend of significantly increased annual NTD mortality rate per 10,000 living born infants since 1990 (Ramirez-Espitia et al. 2003).

The investigation of *MTHFR* 677TT in NTDs risk has been well documented with studies showing a significant association of the genotype with NTDs risk (Yan et al. 2012; Harisha et al. 2010; Grandone et al. 2006) and others not supporting the association or revealing a complex mechanism by which folate intake, parental and infant genotype interplay (Johnson et al. 1999; Shaw et al. 1998; Shields et al. 1999).

In this analysis, we observed that the 1000GP Han Chinese population data *MTHFR* 677TT had a lower frequency (15 %) compared with a large study recently published and being included also in ALFRED, which included >15,000 adults of Han nationality from 10 regions in China (Yang et al. 2013). Older reports on the *MTHFR* 677TT have also reported lower frequencies compared with Yang et al. (Wilcken et al. 2003). This may suggest that important differences in the frequency distribution of functional variants within populations can be more accurately described when more comprehensive analyses are done. Considering the relevance of the *MTHFR* C677T polymorphism for public health, these results highlight the potential need of more detailed studies in different populations to better understand the worldwide frequency distribution of this and also other clinically relevant variants.

Though a previous study has reported a decreased proportion of *MTHFR* 677TT in female newborns (Rozen et al. 1999), proposing that gender effects exist, our study has not identified a large gender effect for any of the studied variants, in accordance with the previous study in the Han Chinese (Yang et al. 2013).

Our study provides evidence that functional variants other than the *MTHFR* C677T and relevant to the folate-mediated one-carbon metabolism are also specifically enriched in populations, and this can be relevant for informing public health policy but also supporting or generating hypotheses related to their potential role in selection processes. For example, the less studied *FOLH1* T484C and *TCN2* C77G risk variants were found more abundant in AFR and ASIA populations, respectively (Gueant et al. 2007), than in the rest of the populations included in our study. Interestingly, both proteins are involved in the intestinal absorption of factors (folate and B_12_) with a pivotal role in the homocysteine metabolic pathway and the functional variants are thought to impair their function (Gueant et al. 2007; Guo et al. 2013). There are direct associations of these SNPs with NTDs (Guo et al. 2013; Gueant-Rodriguez et al. 2003; Martinelli et al. 2006) and autism (Martinelli et al. 2006). Plausible hypotheses explaining their population distribution could be that they may be part of the selective pressure process or providing a survival advantage. As an example, in CC individuals for the *TCN2* C77G allele, tHcy levels seem to be more responsive to dietary B_12_ or B_12_ supplementation (Garrod et al. 2010); it has been also proposed that this polymorphism could influence the susceptibility to malaria, as higher frequencies of the risk variant were observed in patients with severe malaria (Gueant et al. 2007). *FOLH1* T484C in our study presents almost an exclusively high frequency in populations with an African ancestry; the polymorphism has been associated with plasma folate levels suggesting that genetic frequencies may be under population-specific evolutionary mechanisms related to folate bioavailability (DeVos et al. 2008).

*MTHFR* C677T has also been proposed to interact with environmental factors to confer selective pressure; for example, a correlation with ultraviolet radiation exposure has been proposed, which is thought to partly explain the North-to-South gradient of this risk variant gradient in Europe (Yafei et al. 2012; Cordain and Hickey 2006). A similar phenomenon may be related to the inverse gradient observed for *MTRR* A66C for which higher frequencies of *MTRR* 66C are found in northern Europe (44 % in Finland) compared with southern Europe (19 % in Italy). In the case of the *MTHFR* polymorphisms, several relevant gene–nutrient interactions have been described, for example, *MTHFR* 677T was proposed to confer a selective advantage to populations with higher folate intake, such as south Europeans and Mexicans (Gueant-Rodriguez et al. 2006), and more recently, it was found to be associated with a decrease of both serum folate and cell folate concentrations and an altered mix of circulating folate forms (Farrell et al. 2013). A nutrigenetic association has been reported with vitamin B_2_, a precursor of the flavino-adenine dinucleotide (FAD) and a known cofactor of the *MTHFR* enzyme. In this case, carriers of the TT genotype with suboptimal dietary levels of vitamin B_2_ are benefited by the increased intake of the vitamin improving substantially their tHcy levels and blood pressure (Horigan et al. 2010; McNulty et al. 2006; Wilson et al. 2012, 2013). An improved blood pressure control for *MTHFR* 677TT genotype carriers without being influenced by the type of antihypertensive medication has been demonstrated in pre-hypertensive and hypertensive individuals following B_2_ supplementation (Wilson et al. 2012). In Mexico, high rates of increased blood pressure have been recently documented with ~32 % of the population over 50 years of age having at least stage I systolic hypertension and >47 % being at pre-hypertensive levels (Cortes-Hernandez et al. 2014). Less than 50 % of the populations in Mexico that are aware of their increased blood pressure are estimated to control their blood pressure through either medication or lifestyle interventions (Prince et al. 2012; Basu and Millett 2013). The described interactions between *MTHFR* C677T, diet-related factors such as vitamin B_2_ and control of blood pressure should be considered at least during dietary recommendations, particularly in populations like the Mexican with a high frequency of this risk variant.

In addition to folate and other B vitamins, other dietary factors need to be considered when evaluating the role of the functional variants in the homocysteine metabolic pathway. For example, choline status parameters are influenced by the functional variant *MTHFD1* G1958A, when folate status is low (Ivanov et al. 2009). Carriers of the risk variant were found to be in high risk of developing choline deficiency on a low choline diet compared with carriers of the wild-type variant, having thus an increased choline intake requirement (Kohlmeier et al. 2005). This is particularly important for specific population groups, such as pregnant women due to the proposed role of choline in fetal brain development (Zeisel 2011). Mexican populations in our study have a highly enriched *MTHFD1* 1958AA frequency (~35 %) due to their Amerindian ancestry, as shown in the Nahuas, Totonacas and Zapotecos in our study presenting a *MTHFD1* 1958AA frequency of ~60 %. The need for choline dietary recommendations is therefore more pronounced in these populations.

Finally, studying allelic variants in genes involved in folate metabolism are useful and relevant not only for the nutrigenetics field but also for pharmacogenetics. *MTHFR* 677T may be used to help identify patients with an increased likelihood of methotrexate-related adverse events (Urano et al. 2002; Evans 2002); *SLC19A1* A80G has been associated with decreased methotrexate therapeutic response in individuals with rheumatoid arthritis (Dervieux et al. 2004a).

In summary, our study has provided a comparative analysis of functional variants on key enzymes in the folate and homocysteine metabolic pathway in an admixed population consisting of 1,350 Mexican males and females. By including three Amerindian groups and publicly available data from European, African and East Asian populations, we could provide explanation for the differential distribution of the risk genotypes in Mexico and show their worldwide frequency overview. Our results showed that highly frequent risk variants have population-specific distributions, potentially resulting from either selective pressure or genetic drift. Incorporating these results together with other worldwide reports on folate pathway-related SNPs and dietary exposures could contribute in understanding the progression and prevalence of relevant disorders. Finally, the potential role of some of these variants in conferring genetic risk to public health relevant disorders and traits warrants that these results are considered for public health policy making and to strengthen preventive strategies to improve the definition of specific dietary requirements in relevant populations.

## Electronic supplementary material

Below is the link to the electronic supplementary material.

Supplementary material 1 (DOCX 54 kb)

Supplementary material 2 (TIFF 109384 kb)
