# Supplementary material for: Geographical and ethnic distribution of single nucleotide polymorphisms within genes of the folate/homocysteine pathway metabolism
Source: Genes Nutr. 2014 Aug 9;9(5):421. doi: 10.1007/s12263-014-0421-7 (PMC4172644; doi:10.1007/s12263-014-0421-7)
Supplement: Supplementary file 1 — Supplementary material 1 (DOCX 54 kb) [file 12263_2014_421_MOESM1_ESM.docx]

**Supplementary data**

Supp. Table 1. Single Nucleotide Polymorphisms included in the study and the genotyping call rates in our study for the Mexican Mestizos and the Amerindian groups (Chrom: chromosome).

| **SNPs** | **Amino acid change** | **Chrom** | **NCBI ID** | **Position**  **(Genome Build 37.3)** | **Call rate (Mestizos)** | **Call rate (Amerindians)** |
| --- | --- | --- | --- | --- | --- | --- |
| *FOLH1* T484C | Tyr75His | 11 | rs202676 | 49227620 | 99% | 89% |
| *MTHFD1* G1958A | Arg653Gln | 14 | rs2236225 | 64908845 | 97% | 97% |
| *MTHFR* A1298C | Glu429Ala | 1 | rs1801131 | 11854476 | 97% | 100% |
| *MTHFR* C677T | Ala222Val | 1 | rs1801133 | 11856378 | 97% | 89% |
| *MTR* A2756G | Asp919Gly | 1 | rs1805087 | 237048500 | 99% | 99% |
| *MTRR* A66G | Met22Ile | 5 | rs1801394 | 7870973 | 98% | 89% |
| *SLC19A1* A80G | His27Arg | 21 | rs1051266 | 46957794 | 99% | 89% |
| *TCN2* C77G | Pro259Arg | 22 | rs1801198 | 31011610 | 95% | 89% |

Supp. Table 2. Genotype distribution of *FOLH1* T484C (GUE: Guerrero, GUA: Guanajuato, SON: Sonora, VER: Veracruz, YUC: Yucatan, ZAC: Zacatecas, MEX: All Mexican Mestizos, NAH: Nahuas, ZAP: Zapotecos, TOT: Totonacas, AMI: Amerindians, EUR: 1000GP Europeans, AFR: 1000GP Africans, ASIA: 1000GP East Asians, MAF: Minor Allele Frequency, HWE: Hardy-Weinberg Equilibrium).

|  | ***FOLH1 T484C counts*** | | | | ***FOLH1 T484C frequency*** | | | **MAF** | **HWE test** | |
| --- | --- | --- | --- | --- | --- | --- | --- | --- | --- | --- |
|  | **TT** | **CT** | **CC** | **Total** | **TT** | **CT** | **CC** | **C** | ***χ^2^*** | **p value** |
| GUE | 98 | 78 | 7 | 183 | 0.54 | 0.43 | 0.04 | 0.25 | 3.21 | 0.07 |
| GUA | 85 | 87 | 12 | 184 | 0.46 | 0.47 | 0.07 | 0.30 | 2.75 | 0.09 |
| SON | 108 | 65 | 11 | 184 | 0.59 | 0.35 | 0.06 | 0.24 | 0.08 | 0.76 |
| VER | 92 | 76 | 16 | 184 | 0.50 | 0.41 | 0.09 | 0.29 | 0.003 | 0.95 |
| YUC | 97 | 73 | 5 | 175 | 0.55 | 0.42 | 0.03 | 0.24 | 4.1 | 0.05 |
| ZAC | 96 | 81 | 7 | 184 | 0.52 | 0.44 | 0.04 | 0.26 | 4.1 | 0.04 |
| MEX | 576 | 460 | 58 | 1094 | 0.53 | 0.42 | 0.05 | 0.26 | 7.7 | 0.005 |
| NAH | 84 | 57 | 8 | 149 | 0.56 | 0.38 | 0.05 | 0.24 | 0.17 | 0.67 |
| ZAP | 23 | 15 | 6 | 44 | 0.52 | 0.34 | 0.14 | 0.31 | 1.73 | 0.18 |
| TOT | 13 | 10 | 2 | 25 | 0.52 | 0.40 | 0.08 | 0.28 | 0.001 | 0.96 |
| AMI | 120 | 82 | 16 | 218 | 0.55 | 0.38 | 0.07 | 0.26 | 0.15 | 0.70 |
| EUR | 229 | 126 | 24 | 379 | 0.60 | 0.33 | 0.06 | 0.23 | 1.37 | 0.24 |
| AFR | 27 | 88 | 70 | 185 | 0.15 | 0.48 | 0.38 | 0.62 | 0.006 | 0.94 |
| ASIA | 127 | 121 | 38 | 286 | 0.44 | 0.42 | 0.13 | 0.34 | 1.14 | 0.28 |

Supp. Table 3. Genotype distribution of *TCN2* C77G (GUE: Guerrero, GUA: Guanajuato, SON: Sonora, VER: Veracruz, YUC: Yucatan, ZAC: Zacatecas, MEX: All Mexican Mestizos, NAH: Nahuas, ZAP: Zapotecos, TOT: Totonacas, EUR: 1000GP Europeans, AFR: 1000GP Africans, ASIA: 1000GP East Asians, MAF: Minor Allele Frequency, HWE: Hardy-Weinberg Equilibrium).

|  | ***TCN2 C77G counts*** | | | | ***TCN2 C77G frequency*** | | | **MAF** | **HWE test** | |
| --- | --- | --- | --- | --- | --- | --- | --- | --- | --- | --- |
|  | **CC** | **GC** | **GG** | **Total** | **CC** | **GC** | **GG** | **G** | ***χ^2^*** | **P value** |
| GUE | 86 | 71 | 14 | 171 | 0.50 | 0.42 | 0.08 | 0.29 | 0.014 | 0.9 |
| GUA | 74 | 71 | 27 | 172 | 0.43 | 0.41 | 0.16 | 0.36 | 1.99 | 0.16 |
| SON | 73 | 84 | 20 | 177 | 0.41 | 0.47 | 0.11 | 0.35 | 0.32 | 0.57 |
| VER | 75 | 75 | 23 | 173 | 0.43 | 0.43 | 0.13 | 0.35 | 0.38 | 0.53 |
| YUC | 73 | 76 | 16 | 165 | 0.44 | 0.46 | 0.10 | 0.33 | 0.35 | 0.55 |
| ZAC | 72 | 84 | 19 | 175 | 0.41 | 0.48 | 0.11 | 0.35 | 0.57 | 0.45 |
| MEX | 453 | 461 | 119 | 1033 | 0.44 | 0.45 | 0.12 | 0.34 | 0.01 | 0.91 |
| NAH | 72 | 60 | 17 | 149 | 0.48 | 0.40 | 0.11 | 0.32 | 0.68 | 0.4 |
| ZAP | 23 | 13 | 8 | 44 | 0.52 | 0.30 | 0.18 | 0.33 | 4.83 | 0.03 |
| TOT | 10 | 13 | 0 | 23 | 0.43 | 0.57 | 0.00 | 0.28 | 3.57 | 0.06 |
| AMI | 105 | 86 | 25 | 216 | 0.49 | 0.40 | 0.12 | 0.31 | 1.28 | 0.26 |
| EUR | 126 | 178 | 75 | 379 | 0.33 | 0.47 | 0.20 | 0.43 | 0.71 | 0.39 |
| AFR | 121 | 56 | 8 | 185 | 0.65 | 0.30 | 0.04 | 0.19 | 0.21 | 0.64 |
| ASIA | 57 | 147 | 82 | 286 | 0.20 | 0.51 | 0.29 | 0.54 | 0.37 | 0.54 |

Supp. Table 4. Genotype distribution of *SLC19A1* A80G. (GUE: Guerrero, GUA: Guanajuato, SON: Sonora, VER: Veracruz, YUC: Yucatan, ZAC: Zacatecas, MEX: All Mexican Mestizos, NAH: Nahuas, ZAP: Zapotecos, TOT: Totonacas, EUR: 1000GP Europeans, AFR: 1000GP Africans, ASIA: 1000GP East Asians, MAF: Minor Allele Frequency, HWE: Hardy-Weinberg Equilibrium).

|  | ***SLC19A1 A80G counts*** | | | | ***SLC19A1 A80G frequency*** | | | **MAF** | **HWE test** | |
| --- | --- | --- | --- | --- | --- | --- | --- | --- | --- | --- |
|  | **AA** | **AG** | **GG** | **Total** | **AA** | **AG** | **GG** | **G** | ***χ^2^*** | **P value** |
| GUE | 34 | 87 | 62 | 183 | 0.19 | 0.48 | 0.34 | 0.58 | 0.18 | 0.72 |
| GUA | 34 | 92 | 58 | 184 | 0.18 | 0.50 | 0.32 | 0.57 | 0.05 | 0.81 |
| SON | 38 | 87 | 59 | 184 | 0.21 | 0.47 | 0.32 | 0.56 | 0.32 | 0.57 |
| VER | 30 | 84 | 70 | 184 | 0.16 | 0.46 | 0.38 | 0.61 | 0.32 | 0.57 |
| YUC | 28 | 89 | 60 | 177 | 0.16 | 0.50 | 0.34 | 0.59 | 0.27 | 0.59 |
| ZAC | 36 | 87 | 60 | 183 | 0.20 | 0.48 | 0.33 | 0.57 | 0.19 | 0.65 |
| MEX | 200 | 526 | 369 | 1095 | 0.18 | 0.48 | 0.34 | 0.58 | 0.27 | 0.6 |
| NAH | 30 | 79 | 39 | 148 | 0.20 | 0.53 | 0.26 | 0.53 | 0.75 | 0.38 |
| ZAP | 8 | 25 | 11 | 44 | 0.18 | 0.57 | 0.25 | 0.53 | 0.88 | 0.34 |
| TOT | 4 | 12 | 8 | 24 | 0.17 | 0.50 | 0.33 | 0.58 | 0.02 | 0.88 |
| AMI | 42 | 116 | 58 | 216 | 0.19 | 0.54 | 0.27 | 0.54 | 0.02 | 1.38 |
| EUR | 75 | 180 | 124 | 379 | 0.20 | 0.47 | 0.33 | 0.56 | 0.44 | 0.5 |
| AFR | 89 | 80 | 16 | 185 | 0.48 | 0.43 | 0.09 | 0.30 | 0.11 | 0.74 |
| ASIA | 78 | 147 | 61 | 286 | 0.27 | 0.51 | 0.21 | 0.47 | 0.28 | 0.59 |

Supp. Table 5. Genotype distribution of *MTR A2756G*.(GUE: Guerrero, GUA: Guanajuato, SON: Sonora, VER: Veracruz, YUC: Yucatan, ZAC: Zacatecas, MEX: All Mexican Mestizos, NAH: Nahuas, ZAP: Zapotecos, TOT: Totonacas, EUR: 1000GP Europeans, AFR: 1000GP Africans, ASIA: 1000GP East Asians, MAF: Minor Allele Frequency, HWE: Hardy-Weinberg Equilibrium).

|  | ***MTR A2756G counts*** | | | | ***MTR A2756G frequency*** | | | **MAF** | **HWE test** | |
| --- | --- | --- | --- | --- | --- | --- | --- | --- | --- | --- |
|  | **AA** | **GA** | **GG** | **Total** | **AA** | **GA** | **GG** | **G** | ***χ^2^*** | **P value** |
| GUE | 123 | 56 | 4 | 183 | 0.67 | 0.31 | 0.02 | 0.17 | 0.67 | 0.41 |
| GUA | 135 | 41 | 8 | 184 | 0.73 | 0.22 | 0.04 | 0.15 | 4.07 | 0.043 |
| SON | 125 | 58 | 1 | 184 | 0.68 | 0.32 | 0.01 | 0.16 | 4.42 | 0.035 |
| VER | 123 | 52 | 9 | 184 | 0.67 | 0.28 | 0.05 | 0.19 | 1.25 | 0.26 |
| YUC | 124 | 47 | 6 | 177 | 0.70 | 0.27 | 0.03 | 0.17 | 0.34 | 0.55 |
| ZAC | 127 | 51 | 6 | 184 | 0.69 | 0.28 | 0.03 | 0.17 | 0.09 | 0.75 |
| MEX | 757 | 305 | 34 | 1096 | 0.69 | 0.28 | 0.03 | 0.17 | 0.23 | 0.62 |
| NAH | 88 | 72 | 12 | 172 | 0.51 | 0.42 | 0.07 | 0.28 | 0.28 | 0.59 |
| ZAP | 34 | 14 | 0 | 48 | 0.71 | 0.29 | 0.00 | 0.15 | 1.39 | 0.23 |
| TOT | 16 | 6 | 2 | 24 | 0.67 | 0.25 | 0.08 | 0.21 | 1.4 | 0.23 |
| AMI | 138 | 92 | 14 | 244 | 0.57 | 0.38 | 0.06 | 0.25 | 0.07 | 0.79 |
| EUR | 259 | 114 | 6 | 379 | 0.68 | 0.30 | 0.02 | 0.17 | 2.74 | 0.09 |
| AFR | 90 | 74 | 21 | 185 | 0.49 | 0.40 | 0.11 | 0.31 | 0.92 | 0.33 |
| ASIA | 220 | 58 | 8 | 286 | 0.77 | 0.20 | 0.03 | 0.13 | 2.84 | 0.09 |

Supp. Table 6. Genotype distribution of *MTRR* A66G*.* (GUE: Guerrero, GUA: Guanajuato, SON: Sonora, VER: Veracruz, YUC: Yucatan, ZAC: Zacatecas, MEX: All Mexican Mestizos, NAH: Nahuas, ZAP: Zapotecos, TOT: Totonacas, EUR: 1000GP Europeans, AFR: 1000GP Africans, ASIA: 1000GP East Asians, MAF: Minor Allele Frequency, HWE: Hardy-Weinberg Equilibrium).

|  | ***MTRR A66G counts*** | | | | ***MTRR A66G frequency*** | | | **MAF** | **HWE test** | |
| --- | --- | --- | --- | --- | --- | --- | --- | --- | --- | --- |
|  | **AA** | **GA** | **GG** | **Total** | **AA** | **GA** | **GG** | **G** | ***χ^2^*** | **P value** |
| GUE | 137 | 40 | 7 | 184 | 0.74 | 0.22 | 0.04 | 0.15 | 3.2 | 0.07 |
| GUA | 113 | 58 | 10 | 181 | 0.62 | 0.32 | 0.06 | 0.22 | 0.49 | 0.48 |
| SON | 82 | 76 | 21 | 179 | 0.46 | 0.42 | 0.12 | 0.33 | 0.27 | 0.6 |
| VER | 115 | 59 | 8 | 182 | 0.63 | 0.32 | 0.04 | 0.21 | 0.01 | 0.9 |
| YUC | 103 | 58 | 15 | 176 | 0.59 | 0.33 | 0.09 | 0.25 | 2.58 | 0.1 |
| ZAC | 94 | 76 | 11 | 181 | 0.52 | 0.42 | 0.06 | 0.27 | 0.72 | 0.4 |
| MEX | 644 | 367 | 72 | 1083 | 0.59 | 0.34 | 0.07 | 0.24 | 3.9 | 0.05 |
| NAH | 133 | 16 | 0 | 149 | 0.89 | 0.11 | 0.00 | 0.05 | 0.48 | 0.49 |
| ZAP | 41 | 3 | 0 | 44 | 0.93 | 0.07 | 0.00 | 0.03 | 0.05 | 0.81 |
| TOT | 20 | 5 | 0 | 25 | 0.80 | 0.20 | 0.00 | 0.10 | 0.3 | 0.57 |
| AMI | 194 | 24 | 0 | 218 | 0.89 | 0.11 | 0.00 | 0.06 | 0.74 | 0.39 |
| EUR | 93 | 161 | 125 | 379 | 0.25 | 0.42 | 0.33 | 0.54 | 7.89 | 0.004 |
| AFR | 93 | 81 | 11 | 185 | 0.50 | 0.44 | 0.06 | 0.28 | 1.49 | 0.22 |
| ASIA | 159 | 103 | 24 | 286 | 0.56 | 0.36 | 0.08 | 0.26 | 1.53 | 0.21 |

Supp. Table 7. Genotype distribution of *MTHFR* A1298C*.* (GUE: Guerrero, GUA: Guanajuato, SON: Sonora, VER: Veracruz, YUC: Yucatan, ZAC: Zacatecas, MEX: All Mexican Mestizos, NAH: Nahuas, ZAP: Zapotecos, TOT: Totonacas, EUR: 1000GP Europeans, AFR: 1000GP Africans, ASIA: 1000GP East Asians, MAF: Minor Allele Frequency, HWE: Hardy-Weinberg Equilibrium).

|  | ***MTHFR A1298C*** | | | | ***MTHFR A1298C frequency*** | | | **MAF** | **HWE test** | |
| --- | --- | --- | --- | --- | --- | --- | --- | --- | --- | --- |
|  | **AA** | **CA** | **CC** | **Total** | **AA** | **CA** | **CC** | **C** | ***χ^2^*** | **P value** |
| GUE | 154 | 29 | 1 | 184 | 0.84 | 0.16 | 0.01 | 0.08 | 0.085 | 0.77 |
| GUA | 135 | 45 | 4 | 184 | 0.73 | 0.24 | 0.02 | 0.14 | 0.012 | 0.91 |
| SON | 100 | 65 | 13 | 178 | 0.56 | 0.37 | 0.07 | 0.26 | 0.29 | 0.58 |
| VER | 129 | 39 | 2 | 170 | 0.76 | 0.23 | 0.01 | 0.13 | 0.25 | 0.61 |
| YUC | 133 | 38 | 4 | 175 | 0.76 | 0.22 | 0.02 | 0.13 | 0.41 | 0.51 |
| ZAC | 128 | 50 | 6 | 184 | 0.70 | 0.27 | 0.03 | 0.17 | 0.167 | 0.68 |
| MEX | 779 | 266 | 30 | 1075 | 0.72 | 0.25 | 0.03 | 0.15 | 1.569 | 0.21 |
| NAH | 163 | 9 | 0 | 172 | 0.95 | 0.05 | 0.00 | 0.03 | 0.124 | 0.72 |
| ZAP | 45 | 4 | 0 | 49 | 0.92 | 0.08 | 0.00 | 0.04 | 0.88 | 0.76 |
| TOT | 16 | 8 | 0 | 24 | 0.67 | 0.33 | 0.00 | 0.17 | 0.96 | 0.32 |
| AMI | 224 | 21 | 0 | 245 | 0.91 | 0.09 | 0.00 | 0.04 | 0.96 | 0.49 |
| EUR | 176 | 162 | 41 | 379 | 0.46 | 0.43 | 0.11 | 0.32 | 0.165 | 0.68 |
| AFR | 129 | 52 | 4 | 185 | 0.70 | 0.28 | 0.02 | 0.16 | 0.219 | 0.64 |
| ASIA | 187 | 89 | 10 | 286 | 0.65 | 0.31 | 0.03 | 0.19 | 0.021 | 0.88 |

Supp. Table 8. Genotype distribution of *MTHFR* C677T (GUE: Guerrero, GUA: Guanajuato, SON: Sonora, VER: Veracruz, YUC: Yucatan, ZAC: Zacatecas, MEX: All Mexican Mestizos, NAH: Nahuas, ZAP: Zapotecos, TOT: Totonacas, EUR: 1000GP Europeans, AFR: 1000GP Africans, ASIA: 1000GP East Asians, MAF: Minor Allele Frequency, HWE: Hardy-Weinberg Equilibrium).

|  | ***MTHFR C677T counts*** | | | | ***MTHFR C677T frequency*** | | | **MAF** | **HWE test** | |
| --- | --- | --- | --- | --- | --- | --- | --- | --- | --- | --- |
|  | **CC** | **TC** | **TT** | **Total** | **CC** | **TC** | **TT** | **T** | ***χ^2^*** | **P value** |
| GUE | 34 | 87 | 60 | 181 | 0.19 | 0.48 | 0.33 | 0.57 | 0.06 | 0.8 |
| GUA | 41 | 98 | 45 | 184 | 0.22 | 0.53 | 0.24 | 0.51 | 0.79 | 0.37 |
| SON | 57 | 80 | 29 | 166 | 0.34 | 0.48 | 0.17 | 0.42 | 0.01 | 0.91 |
| VER | 33 | 95 | 54 | 182 | 0.18 | 0.52 | 0.30 | 0.56 | 0.61 | 0.43 |
| YUC | 46 | 95 | 33 | 174 | 0.26 | 0.55 | 0.19 | 0.46 | 1.67 | 0.195 |
| ZAC | 51 | 89 | 41 | 181 | 0.28 | 0.49 | 0.23 | 0.47 | 0.03 | 0.85 |
| MEX | 262 | 544 | 262 | 1068 | 0.25 | 0.51 | 0.25 | 0.50 | 0.37 | 0.54 |
| NAH | 10 | 52 | 88 | 150 | 0.07 | 0.35 | 0.59 | 0.76 | 0.37 | 0.54 |
| ZAP | 7 | 9 | 26 | 42 | 0.17 | 0.21 | 0.62 | 0.73 | 8.93 | 0.002 |
| TOT | 2 | 14 | 9 | 25 | 0.08 | 0.56 | 0.36 | 0.64 | 1.51 | 0.28 |
| AMI | 19 | 75 | 123 | 217 | 0.09 | 0.35 | 0.57 | 0.74 | 2.28 | 0.13 |
| EUR | 163 | 165 | 51 | 379 | 0.43 | 0.44 | 0.13 | 0.35 | 0.8 | 0.37 |
| AFR | 146 | 39 | 0 | 185 | 0.79 | 0.21 | 0.00 | 0.11 | 2.56 | 0.1 |
| ASIA | 118 | 126 | 42 | 286 | 0.41 | 0.44 | 0.15 | 0.37 | 0.77 | 0.38 |

Supp. Table 9. Genotype distribution of *MTHFD1 G1958A* in the studies populations

(GUE: Guerrero, GUA: Guanajuato, SON: Sonora, VER: Veracruz, YUC: Yucatan, ZAC: Zacatecas, MEX: All Mexican Mestizos, NAH: Nahuas, ZAP: Zapotecos, TOT: Totonacas, EUR: 1000GP Europeans, AFR: 1000GP Africans, ASIA: 1000GP East Asians, MAF: Minor Allele Frequency, HWE: Hardy-Weinberg Equilibrium).

|  | ***MTHFD1 G1958A counts*** | | | | ***MTHFD1 G1958A frequency*** | | | **MAF** | **HWE test** | |
| --- | --- | --- | --- | --- | --- | --- | --- | --- | --- | --- |
|  | **AA** | **GA** | **GG** | **Total** | **AA** | **GA** | **GG** | **A** | ***χ^2^*** | **P value** |
| GUE | 69 | 84 | 31 | 184 | 0.38 | 0.46 | 0.17 | 0.60 | 0.39 | 0.53 |
| GUA | 61 | 84 | 26 | 171 | 0.36 | 0.49 | 0.15 | 0.60 | 0.11 | 0.74 |
| SON | 38 | 97 | 47 | 182 | 0.21 | 0.53 | 0.26 | 0.48 | 0.85 | 0.35 |
| VER | 65 | 83 | 33 | 181 | 0.36 | 0.46 | 0.18 | 0.59 | 0.51 | 0.47 |
| YUC | 68 | 84 | 24 | 176 | 0.39 | 0.48 | 0.14 | 0.63 | 0.06 | 0.8 |
| ZAC | 62 | 84 | 30 | 176 | 0.35 | 0.48 | 0.17 | 0.59 | 0.03 | 0.86 |
| MEX | 363 | 516 | 191 | 1070 | 0.34 | 0.48 | 0.18 | 0.58 | 0.10 | 0.74 |
| NAH | 91 | 56 | 17 | 164 | 0.55 | 0.34 | 0.10 | 0.73 | 3.32 | 0.07 |
| ZAP | 31 | 17 | 1 | 49 | 0.63 | 0.35 | 0.02 | 0.81 | 0.59 | 0.44 |
| TOT | 15 | 8 | 0 | 23 | 0.65 | 0.35 | 0.00 | 0.83 | 1.01 | 0.31 |
| AMI | 137 | 81 | 18 | 236 | 0.58 | 0.34 | 0.08 | 0.75 | 1.49 | 0.22 |
| EUR | 71 | 183 | 125 | 379 | 0.19 | 0.48 | 0.33 | 0.43 | 0.07 | 0.78 |
| AFR | 7 | 65 | 113 | 185 | 0.04 | 0.35 | 0.61 | 0.21 | 0.39 | 0.53 |
| ASIA | 11 | 112 | 163 | 286 | 0.04 | 0.39 | 0.57 | 0.23 | 2.39 | 0.12 |

Suppl. Table 10. Genotype counts and frequencies divided by gender for the 1104 Mexican Mestizos (MAF:Minor Allele Frequency).

|  | ***MEN*** | | | | | | | ***WOMEN*** | | | | | | |  |
| --- | --- | --- | --- | --- | --- | --- | --- | --- | --- | --- | --- | --- | --- | --- | --- |
|  | ***Counts*** | | | ***Total*** | ***Mean MAF*** | ***Upper*** | ***Lower*** | ***Counts*** | | | ***Total*** | ***Mean MAF*** | ***Upper*** | ***Lower*** | ***P value*** |
| ***FOLH1 T*484C** | 18 | 242 | 285 | 545 | 3.27 | 4.80 | 9.73 | 40 | 218 | 291 | 549 | 7.27 | 0.22 | 6.32 | 0.0006 |
| ***MTHFD1 G*1958A** | 172 | 254 | 104 | 530 | 32.47 | 27.10 | 43.80 | 191 | 262 | 87 | 540 | 35.45 | 24.27 | 40.67 | 0.065 |
| ***MTHFR A*1298C** | 395 | 131 | 10 | 536 | 1.87 | 0.58 | 6.82 | 384 | 135 | 20 | 539 | 3.70 | 0.31 | 4.05 | 0.066 |
| ***MTHFR C*677T** | 118 | 278 | 130 | 526 | 22.28 | 18.54 | 34.56 | 144 | 266 | 132 | 542 | 26.55 | 16.99 | 27.58 | 0.071 |
| ***MTR A*2756G** | 371 | 153 | 21 | 545 | 3.87 | 0.85 | 3.92 | 386 | 152 | 13 | 551 | 2.38 | 1.35 | 6.38 | 0.063 |
| ***MTRR A*66G** | 317 | 194 | 31 | 542 | 5.75 | 3.79 | 11.41 | 327 | 173 | 41 | 541 | 7.60 | 2.94 | 8.56 | 0.07 |
| ***SLC19A1* A80G** | 111 | 253 | 180 | 544 | 33.12 | 31.22 | 37.38 | 89 | 273 | 189 | 551 | 34.30 | 30.48 | 35.75 | 0.025 |
| ***TCN2 C*776G** | 233 | 223 | 58 | 514 | 11.35 | 7.57 | 15.87 | 220 | 238 | 61 | 519 | 11.72 | 8.34 | 14.36 | 0.394 |

Suppl. Figure 1. Comparison of Minor Allele Frequencies (MAF) of the 8 studies SNPs in the 1104 Mexican Mestizos by gender (**P* value>0.0001 for significant differences in genotype frequencies).
